# 3D Printing of Conductive Tissue Engineering Scaffolds Containing Polypyrrole Nanoparticles with Different Morphologies and Concentrations

**DOI:** 10.3390/ma12152491

**Published:** 2019-08-06

**Authors:** Chunyang Ma, Le Jiang, Yingjin Wang, Fangli Gang, Nan Xu, Ting Li, Zhongqun Liu, Yongjie Chi, Xiumei Wang, Lingyun Zhao, Qingling Feng, Xiaodan Sun

**Affiliations:** 1 School of Earth Sciences and Resources, China University of Geosciences (Beijing), Beijing 100083, China; 2 State Key Laboratory of New Ceramics and Fine Processing, School of Materials Science and Engineering, Tsinghua University, Beijing 100084, China; 3 Key Laboratory of Advanced Materials of Ministry of Education of China, School of Materials Science and Engineering, Tsinghua University, Beijing 100084, China

**Keywords:** 3D printing, tissue engineering, nanotechnology, freeze-drying, solvent casting, conductive polymer, 3D scaffold, polypyrrole

## Abstract

Inspired by electrically active tissues, conductive materials have been extensively developed for electrically active tissue engineering scaffolds. In addition to excellent conductivity, nanocomposite conductive materials can also provide nanoscale structure similar to the natural extracellular microenvironment. Recently, the combination of three-dimensional (3D) printing and nanotechnology has opened up a new era of conductive tissue engineering scaffolds exhibiting optimized properties and multifunctionality. Furthermore, in the case of two-dimensional (2D) conductive film scaffolds such as periosteum, nerve membrane, skin repair, etc., the traditional preparation process, such as solvent casting, produces 2D films with defects of unequal bubbles and thickness frequently. In this study, poly-l-lactide (PLLA) conductive scaffolds incorporated with polypyrrole (PPy) nanoparticles, which have multiscale structure similar to natural tissue, were prepared by combining extrusion-based low-temperature deposition 3D printing with freeze-drying. Furthermore, we creatively integrated the advantages of 3D printing and solvent casting and successfully developed a 2D conductive film scaffold with no bubbles, uniform thickness, and good structural stability. Subsequently, the effects of concentration and morphology of PPy nanoparticles on electrical properties and mechanical properties of 3D conductive scaffolds and 2D conductive films scaffolds have been studied, which provided a new idea for the design of both 2D and 3D electroactive tissue engineering scaffolds.

## 1. Introduction

The biomimetic construction of the physiological microenvironment is the design goal of tissue engineering scaffolds. Electrically active tissue breeds the birth of conductive tissue engineering scaffolds. Conductive materials, whose conductive properties allow cell behavior or tissue response to be stimulated by electrical signals [1], can interact with bioelectricity in cells and tissues to enhance biological responses [2]. Many studies have reported the addition of conductive materials to tissue engineering scaffolds to enhance the biological response of scaffolds [3,4]. Common conductive biomaterials include conductive polymers such as polypyrrole (PPy), polythiophene (PEDOT), polyaniline (PANI), carbon nanotubes (CNT), carbon fibers, and graphene [5]. Among these conductive biomaterials, PPy has become one of the most studied conductive materials in tissue engineering due to good cell compatibility, easy preparation, and high conductivity [6]. However, PPy is rigid, insoluble, nondegradable, and not easy to be processed, which makes it difficult to use as a biological scaffold alone. Therefore, it is a good choice to combine other biocompatible and biodegradable biomaterials with them to form nanocomposite conductive materials [7]. In the previous work of our group, PPy nanoparticles were compounded with polylactic acid (PLA) with excellent biocompatibility, and then PPy/PLA composite films with nanofiber structure and conductivity were prepared by electrospinning [8,9]. These conductive films exhibit good biocompatibility with Schwann cells and human umbilical cord mesenchymal stem cells (huMSC) [8,9] and promote the repair of sciatic nerve defects in rats [10,11]. However, the nerve conduits wrapped by conductive films show poor structural stability, especially after implantation in vivo. 

Three-dimensional (3D) printing is a forming technology based on a computer digital model, which constructs three-dimensional structure by the “bottom-up” discrete–cumulative method. It can construct a biomaterial scaffold, which can accurately control the geometry and size. It is a new generation of tissue scaffold preparation method for personalized treatment [12,13]. However, the development of 3D printing tissue engineering scaffolds is limited by materials with both biocompatibility and yield stress and shear thinning rheological properties [14]. Current research has focused on mixing several biocompatible materials to obtain appropriate rheological properties. However, the mixing proportion will have a huge impact on rheological properties, which may result in nonprintable materials. Therefore, the development of biomaterials suitable for printing is a major challenge [15]. At present, some studies have been carried out to prepare conductive tissue engineering scaffolds by adding conductive nanoparticles such as carbon nanotubes, graphene, and Au nanoparticles to polymer matrix for 3D printing [16,17]. Zhang et al. [18] developed a conductive nanoscaffold for nerve repair by mixing amino-functionalized multi-walled carbon nanotubes (MWCNTs) with photosensitizer-containing polyethylene glycol diacrylate polymer (PEGDA) solution under ultrasound and three-dimensional printing by stereolithography. However, there is little research on the composite of PPy nanoparticles and polymer matrix for 3D printing of conductive scaffolds. Furthermore, in the case of two-dimensional (2D) conductive film scaffolds such as periosteum, nerve membrane, skin repair, etc., the traditional preparation process, such as solvent casting, often produces 2D films with defects of unequal bubbles and thickness.

In this study, poly-l-lactide (PLLA) conductive scaffolds incorporated with PPy nanoparticles, which have a multiscale structure similar to natural tissue, were prepared by combining extrusion-based low-temperature deposition 3D printing with freeze-drying. Furthermore, we creatively integrated the advantages of 3D printing and solvent casting and successfully developed a 2D conductive film scaffold with no bubbles, uniform thickness, and good structural stability. Subsequently, the effects of concentration and morphology of PPy nanoparticles on electrical properties and mechanical properties of 3D conductive scaffolds and 2D conductive film scaffolds have been studied, which provided a new idea for the design of both 2D and 3D electroactive tissue engineering scaffolds.

## 2. Materials and Methods 

### 2.1. Synthesis and Characterization of PPy Nanoparticles

The preparation of tubular PPy nanoparticles (T-PPy) refers to the method in literature [19]. In brief, T-PPy was synthesized by using methyl orange as template. To obtain 6 mM methyl orange solution, 0.196g methyl orange (AR, Beijing Chemical Plant) was dissolved in 100 mL deionized water. FeCl_3_·6H_2_0 (MW270.30, chemical reagent Co., Ltd.) and 420 μL of pyrrole monomer (CP, chemical reagent Co., Ltd.) were added successively and stirred for 24 h to form T-PPy. A vacuum suction system with double-layer filter paper was used for filtration, while absolute ethanol (AR, Beijing Chemical Plant) and deionized water (China Agricultural University) were used for repeated cleaning. Finally, it was dried at 60 °C for 24 h before grinding. 

The preparation of spherical PPy nanoparticles (S-PPy) refers to our previous work [8,9]. In brief, before adding 1.59 mL pyrrole monomer (chemical pure CP, Chemical Reagent Co., Ltd., China), 2.3 g polyethylene oxide–polypropylene oxide–polyethylene oxide (P123, Sigma-Aldrich, St. Louis, Missouri, USA) was dissolved in 227.7 mL deionized water. After 30 min of magnetic stirring, 18.65 g FeCl_3_.6H_2_O (MW 270.30, Chemical Reagent Co., Ltd., China) was added to form S-PPy at 18 °C for 6 h. S-PPy were collected by centrifugation and washed repeatedly with deionized water and ethanol. Finally, it was dried at 60 °C for 24 h before grinding. 

The surface morphology of the PPy nanoparticles was observed by scanning electron microscopy (S-450 SEM, Hitachi, Tokyo, Japan). In brief, Conductive tape was pasted on the sample table with a size of about 4 mm × 8 mm. A small sample was taken and pasted on the conductive tape. The sample was dispersed as far as possible without spraying gold. The microstructure of the PPy nanoparticles was observed by transmission electron microscopy (H-800 TEM, Hitachi, Tokyo, Japan). In brief, 1 mg PPy nanoparticle was dissolved in 2 mL absolute ethanol and dispersed by ultrasound for about 15 min to form a uniform suspension. A drop of suspension was dripped on the microgrid, and the water was absorbed by filter paper and completely dried, then TEM was used to observe it. The conductivity of pure PPy was measured by a four-probe tester (SX1944, suzhou telecommunication instrument factory, Suzhou, China). The PPy nanoparticles were prepared into compact wafers, with a diameter of 10 mm and a thickness of 1 mm–2 mm. Specifically, 3 g PPy nanoparticles was put into an agate mortar to grind them thoroughly. Pure compact polypyrrole wafers were prepared by pressing a special steel die with a diameter of 10 mm. Finally, the conductivity of each sample was measured at three different points, and the average value was taken as the measurement value of this sample.

### 2.2. Preparation and Characterization of 3D Printing Ink

Three-dimensional printing ink was formed by dispersing PPy nanoparticles in 1,4-dioxane solution with poly-l-lactide (PLLA) as solute. In order to keep the same quality of the same scaffold after forming, the total mass concentration of PLLA and PPy in the ink used for fixing the scaffold remained unchanged when preparing 3D printing ink. Ink composition and proportion are shown in Table 1 (C_PPy_ = M_PPy_/M_PPy + PLLA_, C_ink_ = M_PPy + PLLA_/V_1,4-dioxane_, “T-PPy” are denoted by “T”, “S-PPy” are denoted by “S”, the quality of T-PPy/S-PPy/PLLA is denoted by m_PPy_(T)/m_PPy_(S)/m_PLLA_, the volume of 1,4-dioxane was denoted by V_1,4-dioxane_). The preparation process is as follows: (1) PPY nanoparticles were dispersed in 1,4-dioxane, according to the ratio shown in the table. The nanoparticles were dispersed uniformly in solvents by ultrasonic oscillation for 20 min. (2) PLLA powder of corresponding quality was added according to the proportion, stirred until PLLA powder was completely dissolved, and then the bubbles were removed by ultrasonic vibration. In order to determine the effects of PPy with different concentration and morphology (spherical or tubular) on the viscosity and shear rheological properties of 3D printing ink, Super Rotary Rheometer (Kinexus, Malvern Instruments Co., Ltd., Malvern, Britain) was used to measure the viscosity of 3D printing ink in the speed range of 10^−1^–10^2^ rad/s.

### 2.3. 3D Printing of 3D Conductive Scaffold and 2D Conductive Film Scaffold

**Scheme 1:**
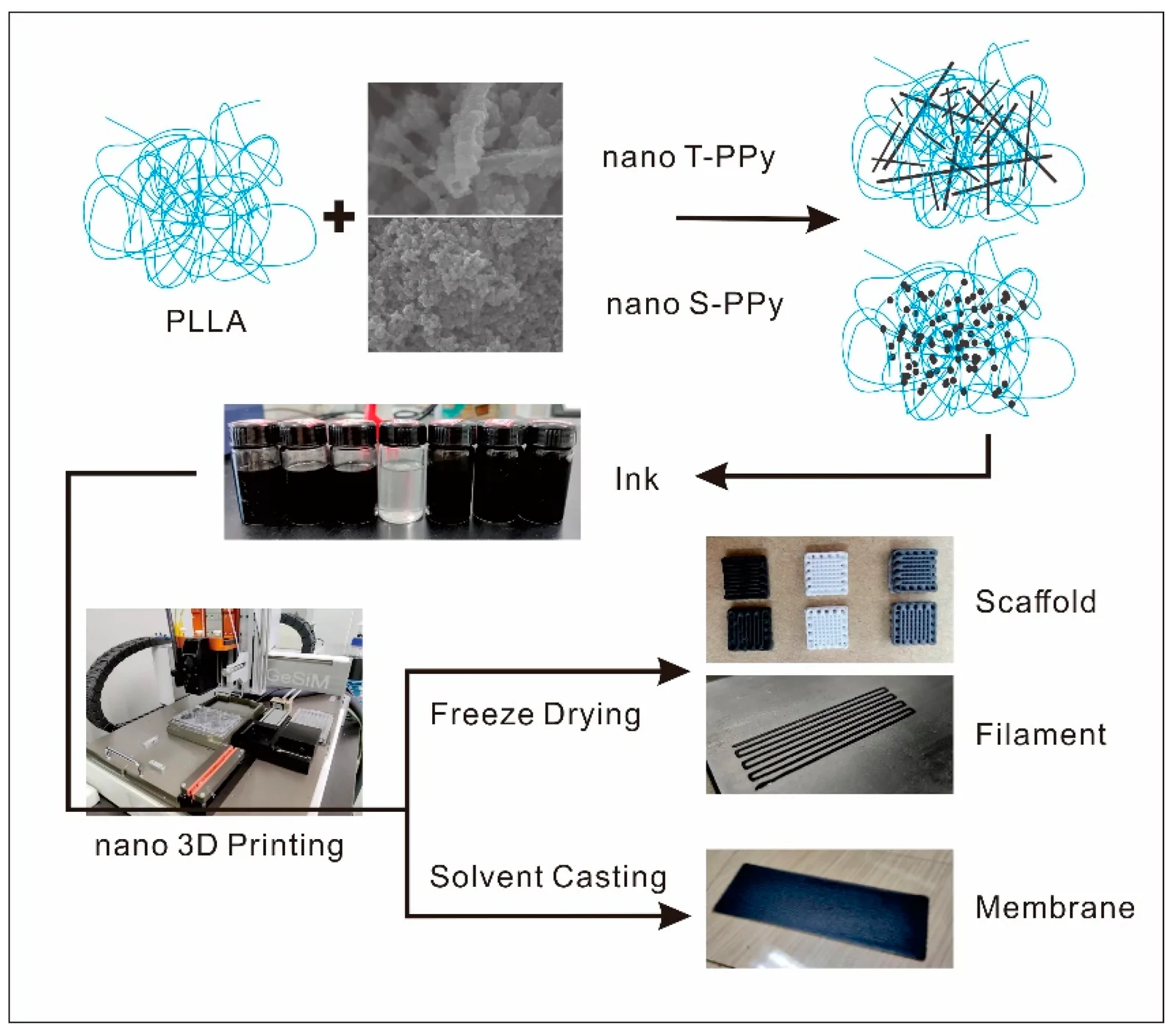
Schematic illustration of the overall experimental design.

A pneumatic extrusion-based 3D printer (BioScaffolder 3.2, Gesim, Germany) was used for 3D printing (Scheme 1). The operation step is to draw a three-dimensional image by using 3D builder software in advance, then import the image into Gesim Robotics software, select the appropriate extrusion and forming method according to the material forming mode with the corresponding printing parameters, and start printing automatic forming. 3D conductive scaffolds were prepared by combining extrusion-based low-temperature deposition 3D printing with freeze-drying. In briefly, the dimension of the printed 3D scaffold was 8 mm × 8 mm × 2 mm and the distance between two adjacent filaments was 1.0 mm. Ink was extruded by a 0.26 mm diameter nozzle at room temperature and received at a condenser at −7 °C. Subsequently, 3D scaffolds were put in a freeze-dryer for 12 h after printing. Furthermore, 2D conductive film scaffolds were prepared by combining extruded 3D printing with solvent casting. In brief, the dimension of the printed 2D film was 30 mm × 15 mm and the distance between two adjacent filaments was 1.2 mm. Ink was extruded by a 0.62 mm diameter nozzle and received at a glass slide at room temperature. Subsequently, 2D film scaffolds were put in the fume hood to dry after printing.

### 2.4. Characterization of 3D Conductive Scaffold and 2D Conductive Film Scaffold

The morphology of 3D conductive scaffold and 2D conductive film scaffold with Cink = 10% was observed by field emission scanning electron microscopy (FE-SEM, Merlin, Zesis, Germany). In order to characterize the electrical properties of 3D conductive scaffolds with different morphologies and concentrations of PPy, the conductivity of conductive scaffolds filament was measured by a digital multimeter (HongDa, DT9202, Shenzhen, China). The conductivity of 2D conductive film scaffold was measured by a four-probe tester (SX1944, suzhou telecommunication instrument factory, Suzhou, China). In order to determine the compressive strength of the conductive scaffold, the electronic universal testing machine was used to compress the scaffold at a compression rate of 1 mm/min, until the thickness of the scaffold was compressed from 2 mm to 1 mm. Similarly, in order to determine the tensile strength of conductive films, the electronic universal testing machine (EUTM, WDW 3020, Changchun, China) was used to stretch the film at the rate of 3 mm/min until the film was broken.

### 2.5. Cytocompatibility Test

About 2000 L929 fibroblasts were cultured on pure PLLA films and films containing spherical or tubular PPy nanoparticles. Before cell culture, the three groups of materials were sterilized with 75% alcohol for 24 h, and then immersed in Dulbecco’s modified Eagle’s medium (DMEM) until equilibrium was reached. After 48 h of incubation in the incubator, CCK8 (produced by Dojindo Laboratories) was added and incubated for 4 h. The absorbance was measured by enzyme label (produced by EnSpire). Cells cultured in the culture plate acted as the blank control group.

### 2.6. Statistical Analysis

Data analysis was carried out by using IBM SPSS Statistics 24. The average value of each group of data was taken to draw a histogram. All quantitative data were expressed by using standard error. Numerical data were analyzed via one-way analysis of variance (ANOVA), with Tukey post-hoc test to determine differences between the experimental groups (PLLA incorporated with nano-PPy) and the control group (pure PLLA), respectively. No significant differences (*p* > 0.05), significant differences (0.01 < *p* < 0.05), significant differences (0.01 < *p* < 0.001), and very significant differences (*p* < 0.001) were denoted by “n.s.”, “*”, “**” and “***”, respectively.

## 3. Results

### 3.1. Characterization of PPy Nanoparticles

**Figure 1:**
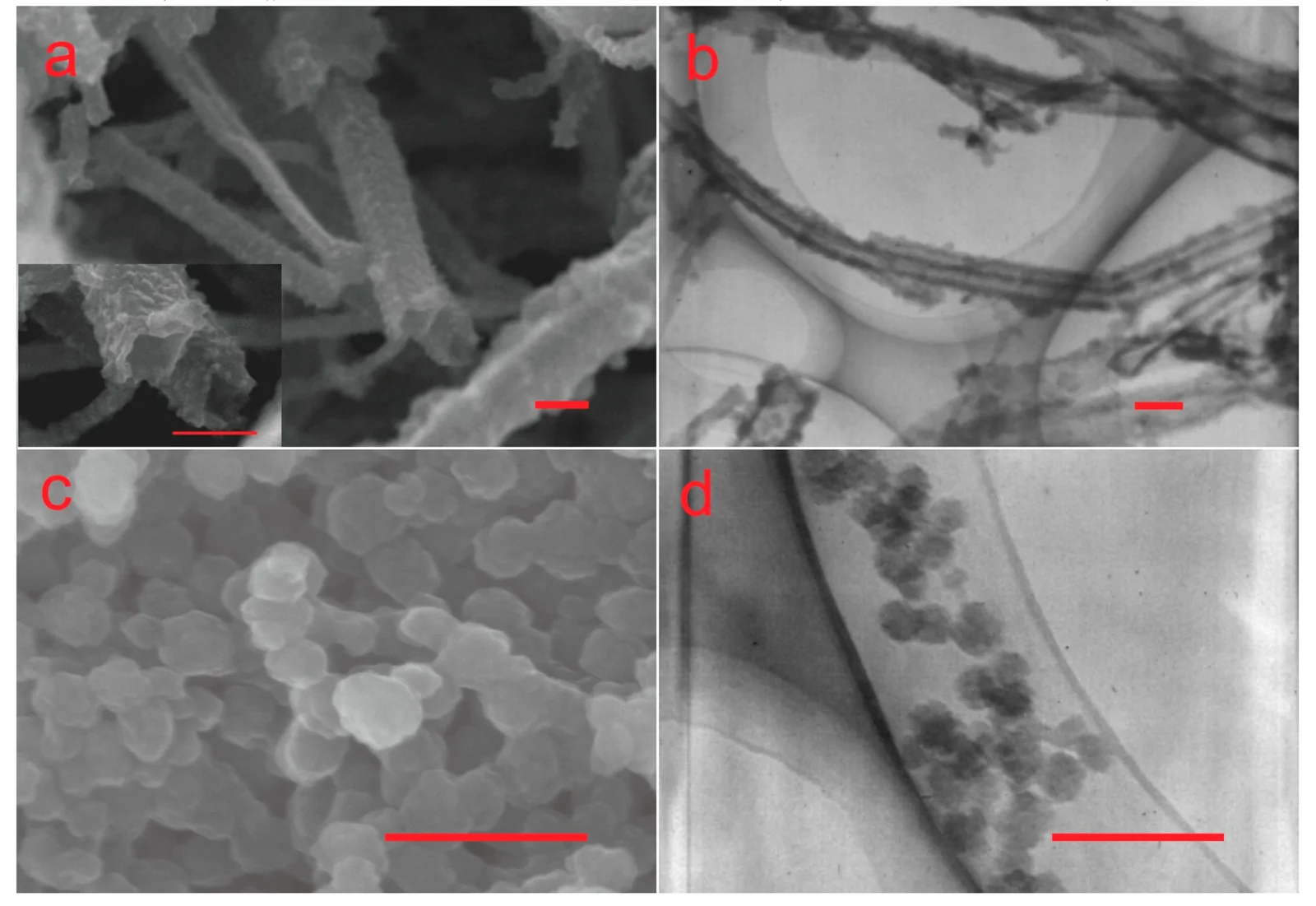
SEM images (**a**) and TEM images (**b**) of tubular PPy nanoparticles (T-PPy), SEM images (**c**) and TEM images (**d**) of spherical PPy nanoparticles (S-PPy). Scale bar is 200 nm.

It can be seen from Figure 1a,b that the morphology of T-PPy was mostly round or square tube, and the square nanotubes had sharp edges and corners; the length was about 10 μm, and the outer diameter was 100–300 nm m; the surface was rough and multi-cross-wound. The conductivity of T-PPy was about 0.48 S/cm, which is in the range of a semiconductor’s conductivity. It can be seen from Figure 1c,d that the diameter of S-PPy was about 50 nm, and the dispersion was good. The conductivity of the powder was about 0.6 S/cm, which is higher than that of the T-PPy.

### 3.2. Characterization of 3D Printing Ink

**Figure 2:**
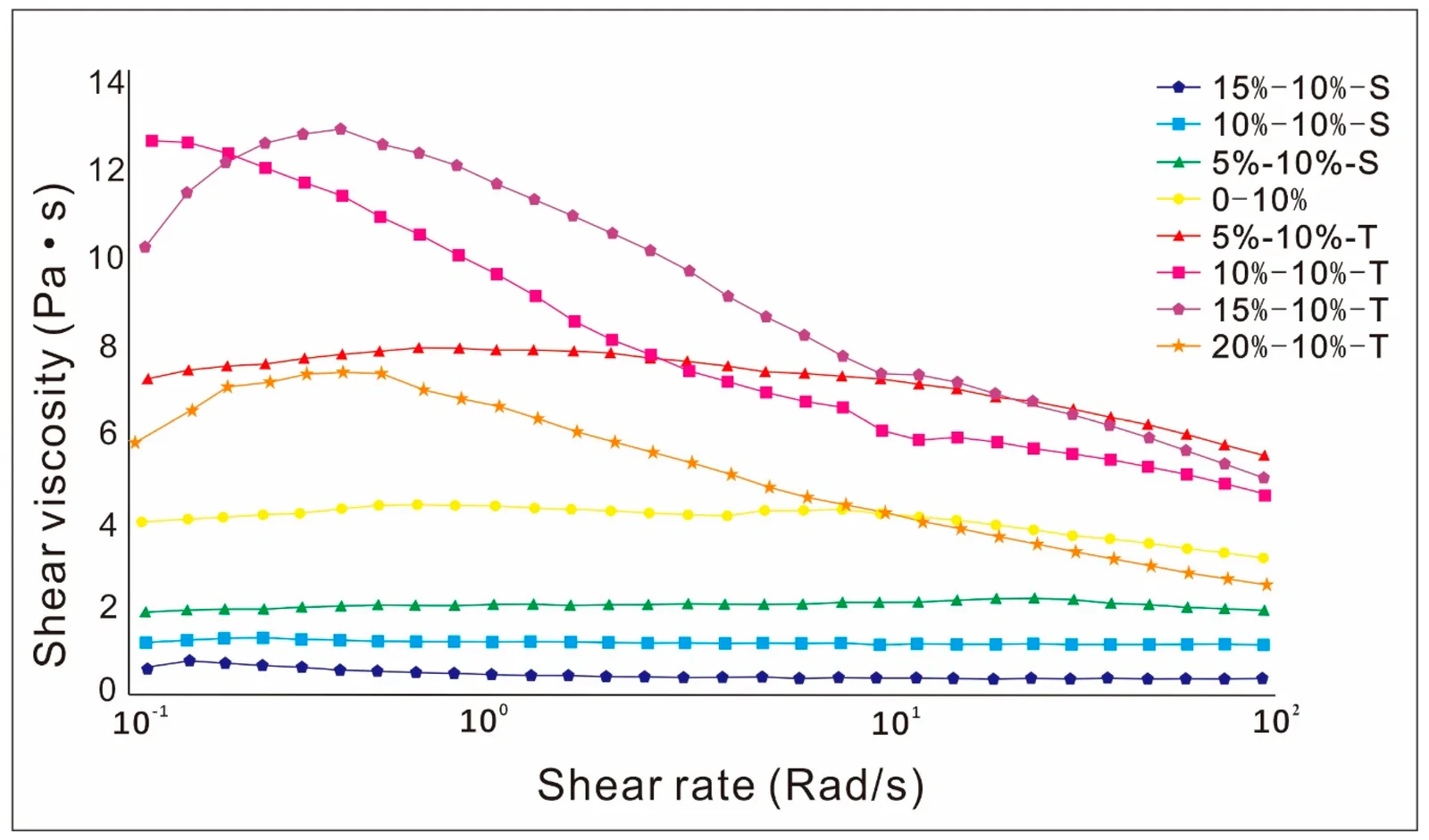
Viscosity curve of 3D printing ink containing polypyrrole (PPy) nanoparticles with different concentration and morphology in the speed range of 0.1–100 rad/s.

As can be seen from Figure 2, the viscosity of the ink decreased gradually with the increase of the concentration of S-PPy. The ink with 10% T-PPy nanoparticles exhibited strong shear-thinning behavior, while the ink with 15% and 20% T-PPy exhibited a strong shear-thickening trend at a low shear rate and shear-thinning behavior at a high shear rate. 

### 3.3. Characterization of 3D Conductive Scaffolds

**Figure 3:**
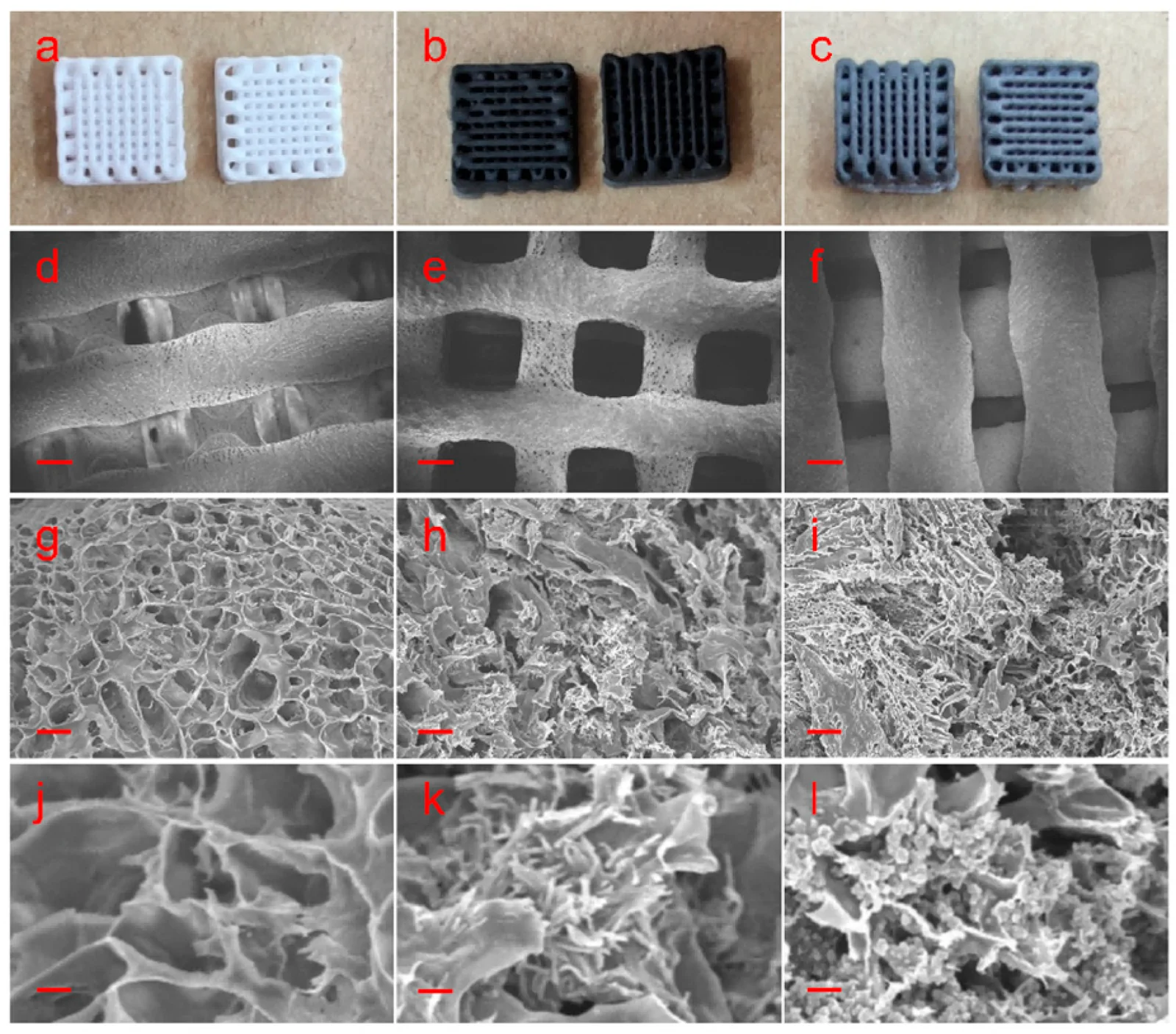
Optical and SEM images of 3D conductive scaffolds containing 10 wt% T-PPy or S-PPy prepared by combining 3D printing and freeze-drying. (**a**–**c**) Optical images of 3D conductive scaffolds (pure, tubular, and spherical in turn), (**d**–**f**) SEM images of the scaffolds at the magnification of 26, scale bar is 200 μm, (pure, tubular, and spherical in turn), (**g**–**i**) SEM images of the scaffolds at the magnification of 1000, scale bar is 10 μm, (**j**–**l**) SEM images of the scaffolds at the magnification of 10,000, scale bar is 1 μm.

From the optical images and low-magnification SEM images of Figure 3a–f, it can be seen that each fiber in the scaffold containing 10 wt% T-PPy or S-PPy was straight and uniform in thickness, and the whole scaffold was square and angular. From the high-magnification SEM images of Figure 3g–l, it can be seen that there was porous structure with uniformly distributed pores in the scaffold. The morphology and size of PPy nanoparticles in the scaffolds were the same as those of pure PPy under electron microscopy. In conclusion, 3D conductive scaffolds with macrocontrollable pores (~100 μm) and microfilament pores (~10 μm) have been successfully fabricated by combining 3D printing with freeze-drying in this study.

**Figure 4:**
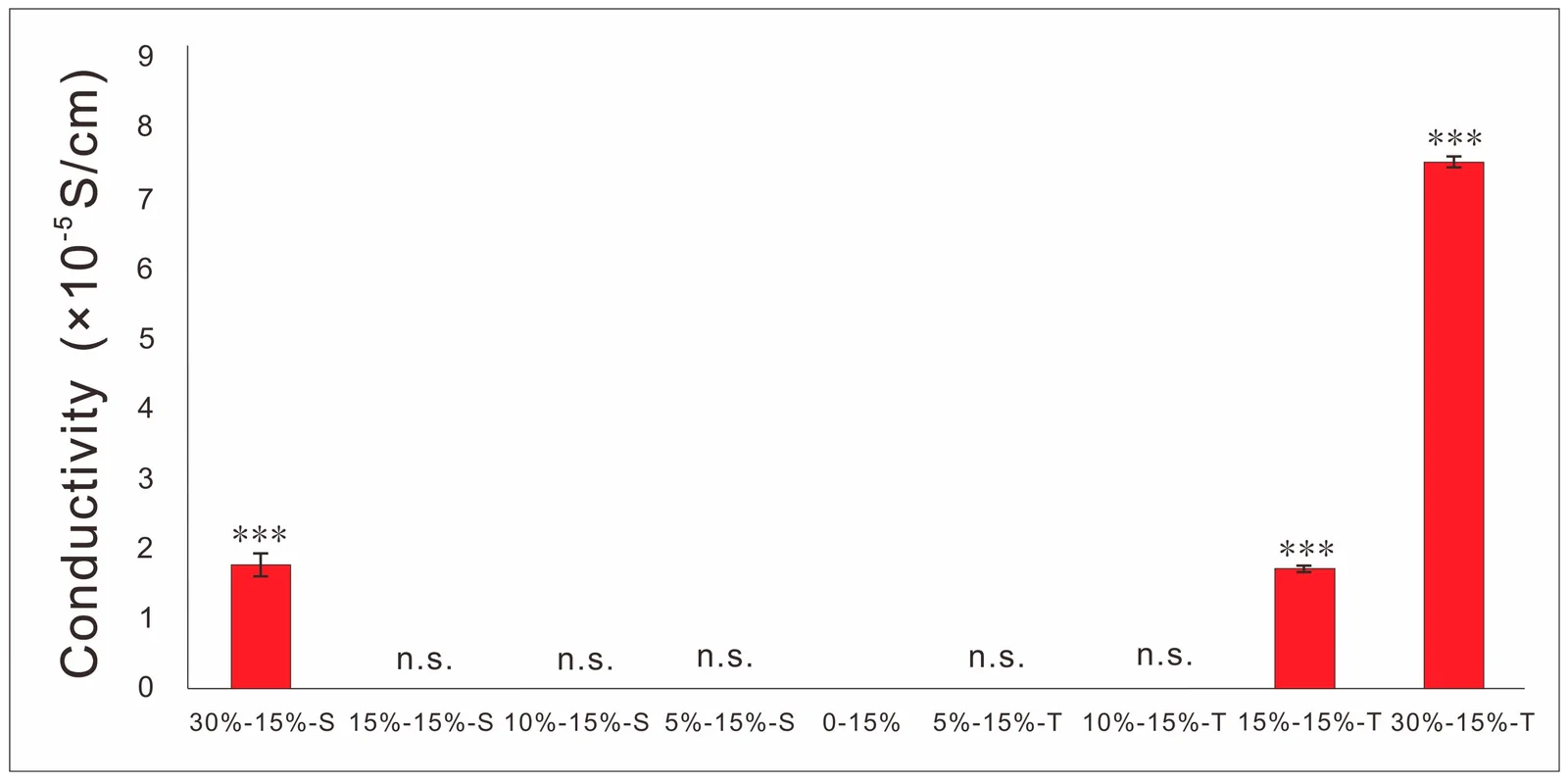
Conductivity of 3D conductive scaffolds.

In order to characterize the electrical properties of 3D conductive scaffolds with different morphologies and concentrations of PPy, the conductivity of conductive scaffolds filament was characterized. It can be seen from Figure 4, the conductivity of 3D scaffolds with the same concentration of T-PPy was higher than that of S-PPy. The 3D conductive scaffold with S-PPy seeped within 15 wt% in 30 wt%, while T-PPy within 10 wt% to 15 wt%.

**Figure 5:**
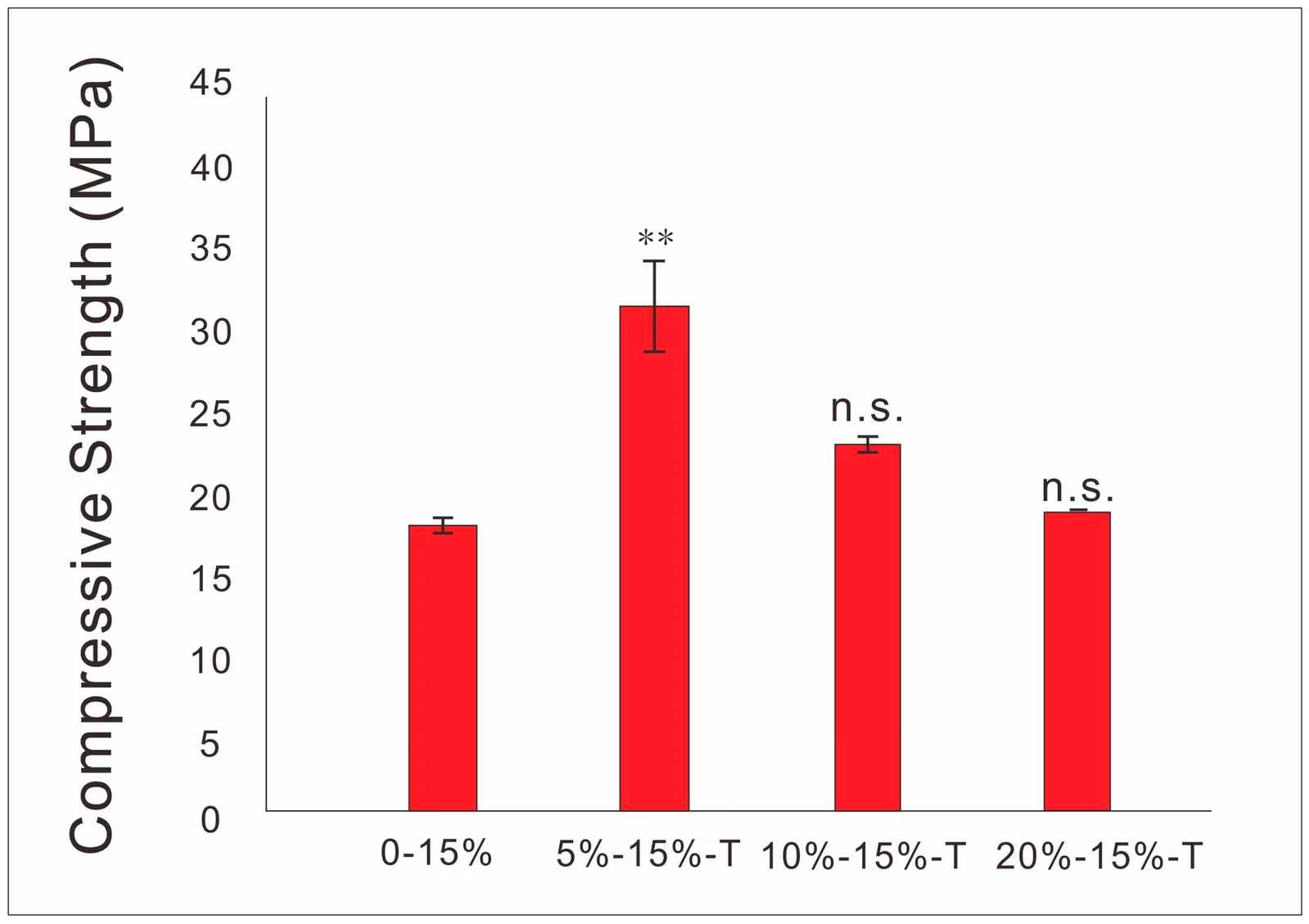
Compressive strength of 3D conductive scaffolds.

It is shown in Figure 5 that the compressive strength of 3D conductive scaffolds increased first, and then decreased with the increase of the content of T-PPy (decrease of the content of PLLA), and reached the highest when the content of T-PPy was 5 wt%.

### 3.4. Characterization of 2D Conductive Films

**Figure 6:**
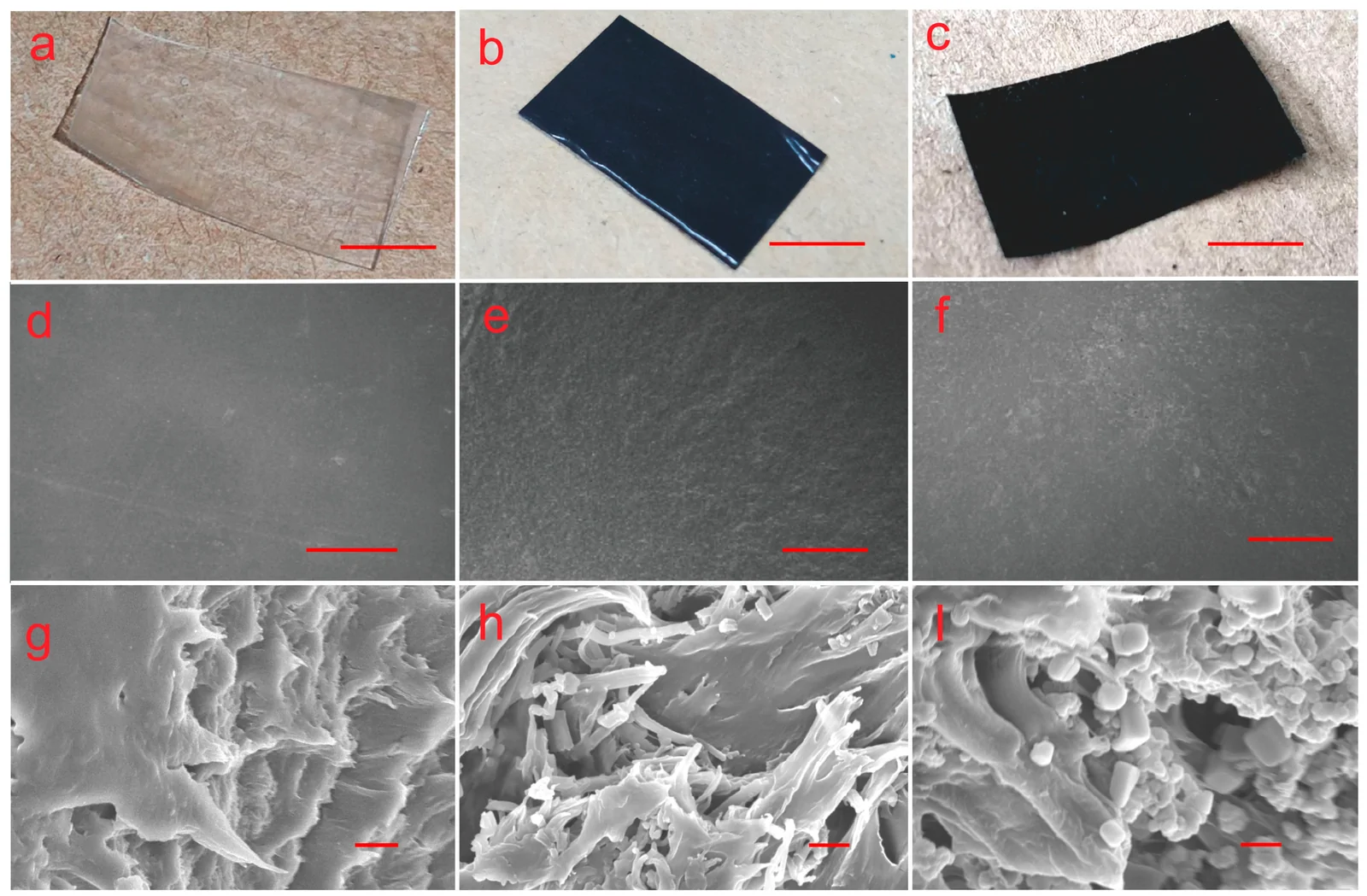
SEM images of pure poly-l-lactide (PLLA) film and films containing 10 wt% T-PPy and S-PPy, (**a**–**c**) physical images of three films, scale bar is 1 cm (pure, T-PPy, and S-PPy, in turn)), (**d**–**f**) SEM images of three films at 104 magnification, scale bar is 200 μm (10 wt% PPy, T-PPy, and S-PPy, in turn), (**g**–**i**) SEM images of three films at 10,000 magnification, and scale bar is 1 μm (10 wt% PPy, T-PPy, and S-PPy, in turn).

As can be seen from Figure 6, the films containing 10 wt% T-PPy or S-PPy prepared by combining 3D printing with solvent casting had smooth surface, uniform material dispersion, no bubbles, uniform thickness, and high structural stability. The morphology of PPy nanoparticles in the scaffold remained good, and the T-PPy did not show obvious breakage. Furthermore, PPy nanoparticles had close contact with PLLA and there were almost no voids. Compared with pure PLLA membrane, the surface of the composite films with nanoparticles was slightly rough.

**Figure 7:**
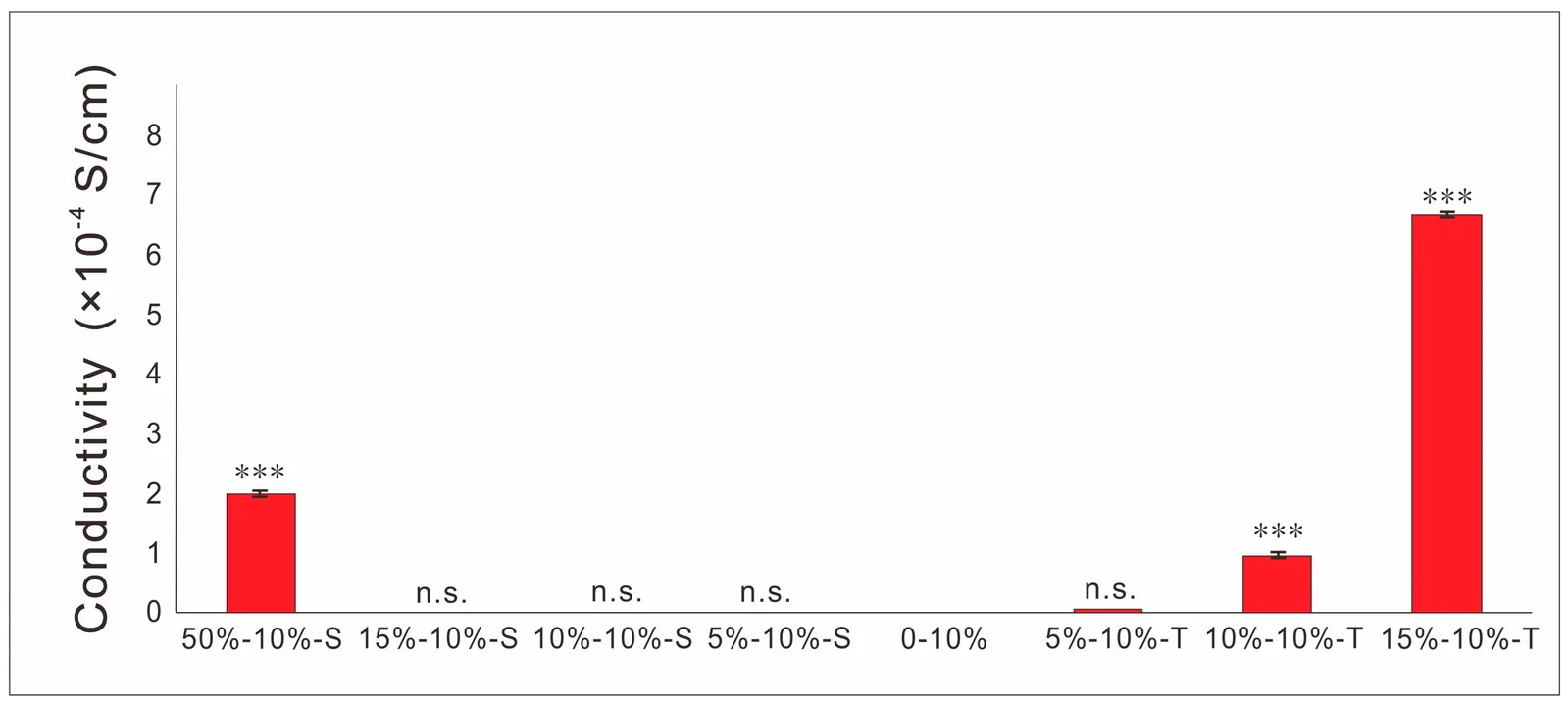
Conductivity of 2D conductive films.

It can be seen from Figure 7 that the conductivity of conductive film containing T-PPy was significantly higher than that of conductive film containing S-PPy at the same concentration. The 2D conductive film with S-PPy seeped in the range of 15 wt% to 50 wt%, while T-PPy in the range of 5 wt% to 10 wt%.

**Figure 8:**
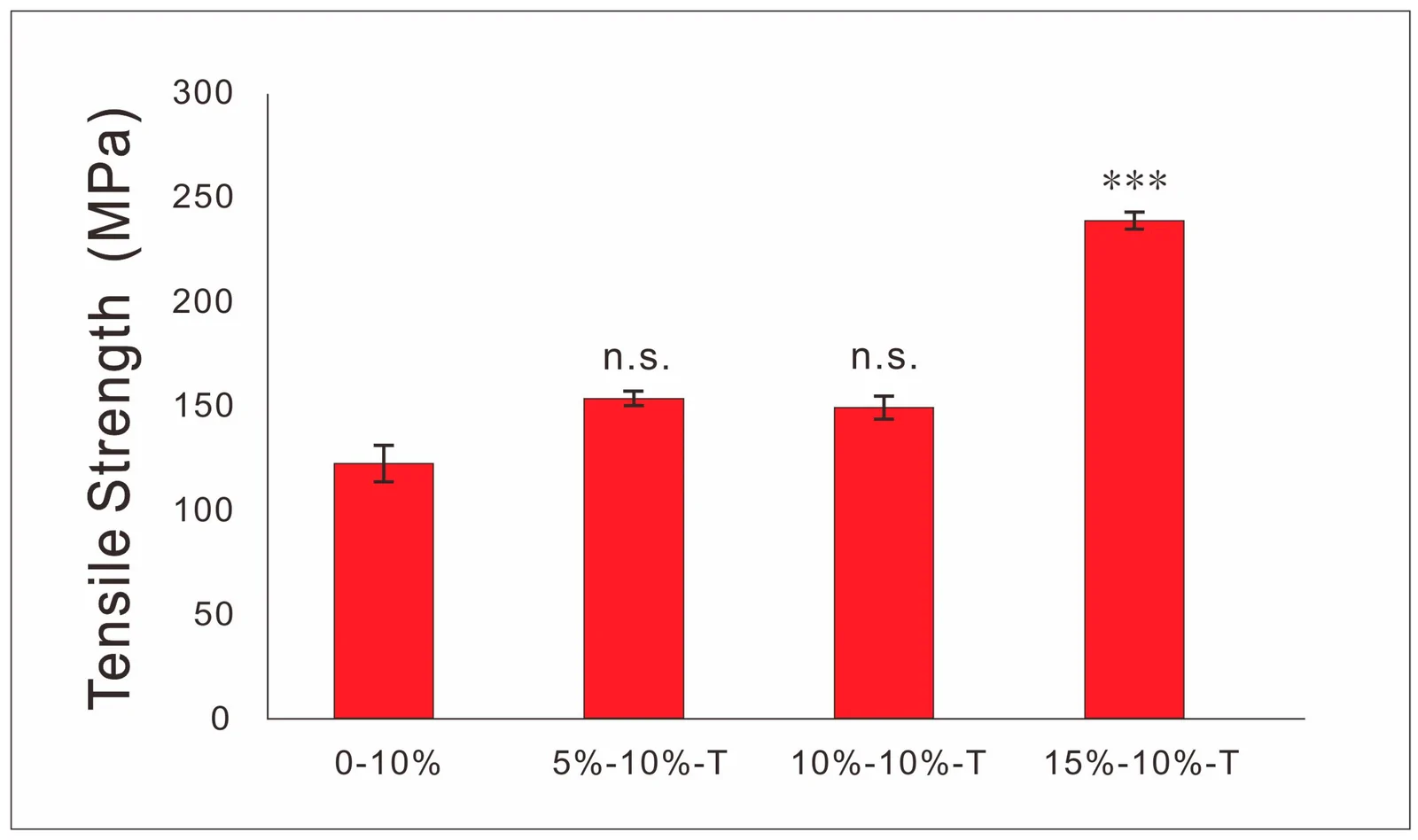
Tensile strength of 2D conductive films.

It can be seen from Figure 8 that the addition of T-PPy improved the tensile strength of the films. The tensile strength of all tested 2D films was more than 100 MPa, and the tensile strength of the 2D conductive films with tubular PPy concentration of 15 wt% reached nearly 250 MPa.

### 3.5. Cytocompatibility Test

**Figure 9:**
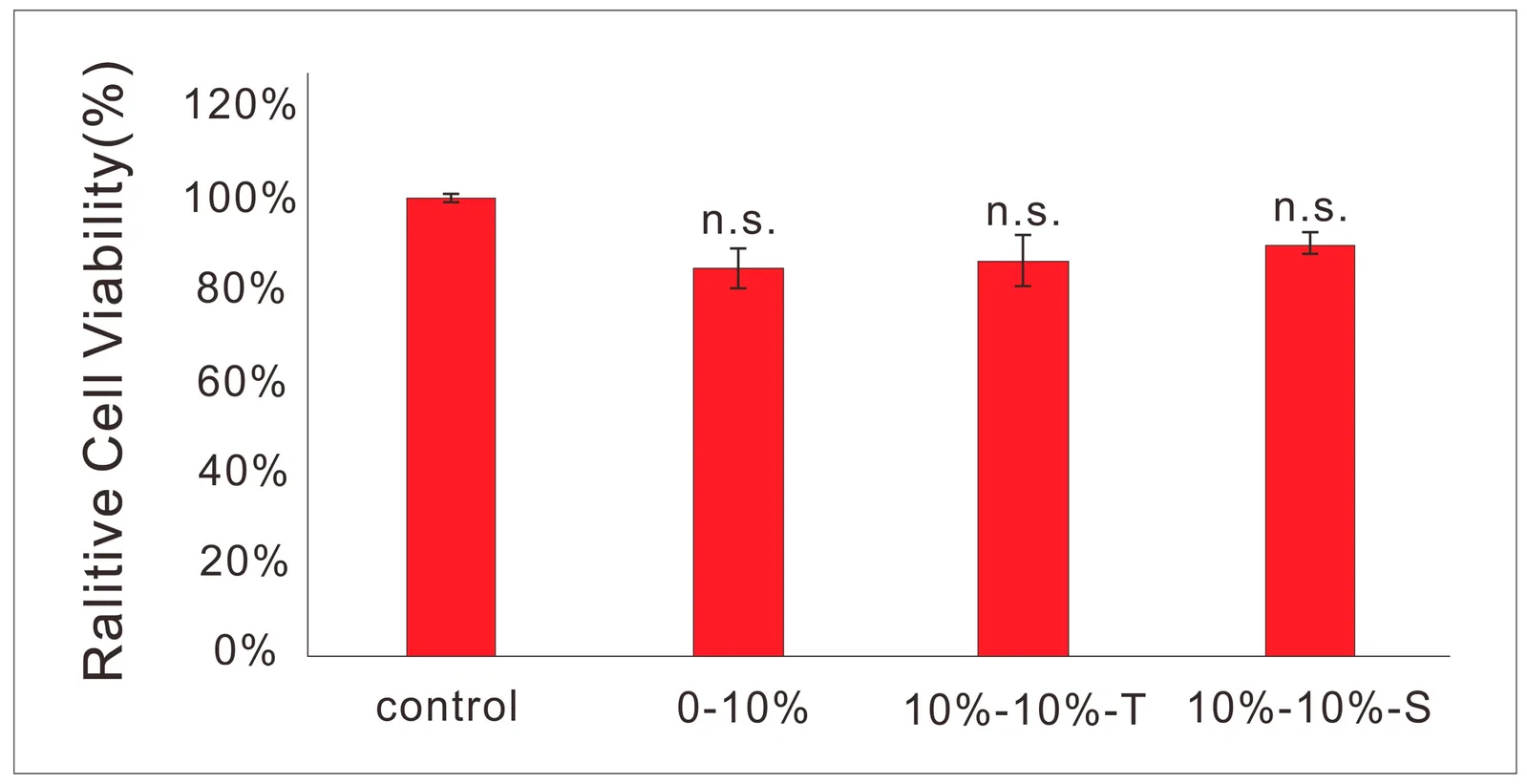
Viability of L929 cells cultured on pure PLLA films and films containing spherical or tubular PPy nanoparticles, compared with blank control group.

It can be seen from Figure 9 that after two days of culture, the cell viability of pure PLLA material, composite material containing tube, or spherical PPy was higher than 80%, and there was no significant difference between the three groups of materials and blank control group.

## 4. Discussion

Inspired by electrically active tissues, conductive materials have been extensively studied and developed for electrically active tissue engineering scaffolds. Due to the limitation of mechanical properties of conductive materials, nanocomposite conductive materials are usually prepared by compounding nanoparticles with polymer matrix. In addition to excellent conductivity, nanocomposite conductive materials can also provide a nanoscale structure similar to the natural extracellular microenvironment. Recently, 3D printing has attracted much attention in the field of tissue engineering due to customizable formability and its ability to produce complex structures [20]. Domingos et al. [21] fabricated bone tissue engineering scaffolds by 3D printing and studied the effects of polycaprolactone (PCL) scaffolds with nano-hydroxyapatite (nHA) and micron-hydroxyapatite (mHA) particles on the adhesion and differentiation of mesenchymal stem cells. The results showed that nHA-PCL scaffold promoted more adhesion and differentiation of human mesenchymal stem cells compared with mHA-PCL [21]. The combination of 3D printing and nanotechnology has opened up a new era of conductive tissue engineering scaffolds exhibiting optimized properties and multifunctionality [14]. Huang et al. [22] successfully fabricated PCL scaffolds containing 3 wt% MWCNTs by fused deposition 3D printing technology, which exhibited similar mechanical strength to cancellous bone and improved cell adhesion. However, melt extrusion-based 3D printing technology is difficult to ensure the uniform dispersion of nanoparticles in polymer matrix, which will cause structural heterogeneity, poor mechanical properties, and other defects [23]. Ho [24] et al. successfully fabricated PCL scaffolds containing 5 wt% MWCNTs using low-temperature deposition 3D printing technology. 3D printing ink was prepared by mixing 5 wt% MWCNTs suspension in chloroform with PCL solution, using chloroform as solvent. The results showed that the compressive strength and H9C2 cell proliferation of the scaffold were improved by adding MWCNTs. However, because of the strong volatility of chloroform, the solution composition of 3D printing ink was uncontrollable, which will greatly affect the accuracy and repeatability of 3D printing. In addition, the electrical conductivity and mechanical properties of final 3D printing scaffold depend on the morphology of nanofillers [14]. However, as far as we know, there has been no study on the electrical and mechanical properties of 3D printing scaffolds with different morphologies of conductive nanoparticles. 

In the process of extrusion-based 3D printing, ink viscosity is an important factor affecting printing quality. Especially when using a pressure-driven method for 3D printing, the extrusion speed of ink with different viscosities will vary greatly under the same pressure, which will directly affect the fiber diameter and pore of the scaffold, and even affect the overall forming effect of the scaffold [25,26]. For printing of 2D film, the ink with relatively low viscosity should be selected for printing, because the surface of the film needs to be smoothed by the surface tension of the liquid. However, if the viscosity is too small, the ink will escape everywhere after extrusion, which will affect the formation of the intact film’s normal forming. Therefore, the ink with Cink = 10 wt% was chosen selected as the optimized concentration for printing 2D film in this work. For printing of 3D scaffolds, the ink with relatively high viscosity will prevent collapse after extrusion, which is conducive to the formation of scaffolds. However, if the viscosity is too high, it is difficult to extrude and easy to block the nozzle. So the ink with Cink = 15 wt% was selected as the optimized condition for print 3D scaffolds in this work. Furthermore, studies have shown that ink with shear-thinning behavior may be more appropriate for 3D printing [27]. The shear force of ink in the nozzle is larger than that in the cylinder and after squeezing through the nozzle. Ink with strong shear-thinning behavior has smaller viscosity at the nozzle, and it is not easy to plug the nozzle. After being extruded from the nozzle, the viscosity increases, which can maintain the shape of the filament and is conducive to maintaining the shape of the scaffold. On the contrary, ink with strong shear-thickening behavior has smaller viscosity at the nozzle, and it is prone to plug the nozzle. After being extruded from the nozzle, the viscosity decreases, which cannot maintain the shape of the filament and is not conducive to maintaining the shape of the scaffold. In this study, the composition of solution can be well controlled by using 1,4-dioxane with slow volatility as solvent. The ink viscosity increased firstly, and then decreased with the increase of the concentration of T-PPy, while the total mass concentration of PPy and PLLA remained unchanged. This may be attributed to the reason that adding a small amount of T-PPy can increase the ink viscosity. But when the concentration of T-PPy was too high, the concentration of matrix polymer (PLLA) was relatively low, so the ink viscosity will decrease. It has been reported that nanoparticles with relatively high concentration in the polymer matrix could form “particle clusters” to act as a barrier structure of aggregates so that the viscosity of the system is increased [28]. When the shear stress increased to be higher than the critical point, the disorderly arrangement of “particle clusters” in the system would turn to be orderly so that the viscosity of the system turned to decrease [28]. The shear rheological results showed that 10 wt% T-PPy may be more appropriate for extrusion-based 3D printing. Our previous work showed that the solution viscosity increased slightly with the addition of S-PPy, while the concentration of PLLA remained unchanged [7,8]. However, the ink viscosity increased first, and then decreased with the increase of S-PPy concentration (the total mass concentration of PPy and PLLA was unchanged) in this study. This may be attributed to the fact that the increase of viscosity aroused by the increase of S-PPy concentration could not compensate for the decrease of viscosity aroused by the decrease of PLLA concentration, so the total viscosity showed a downward trend. 

The spatial grid structure with macrocontrollable pores (~100 μm) can be manufactured by extrusion-based 3D printing, and the micropores (~10 μm) at cell-level can be manufactured by freeze-drying technology [29,30,31]. Zhang et al. [28] has successfully fabricated tissue engineering scaffolds with both macroporous spatial grid structure and microporous structure by combining extrusion-based low-temperature deposition 3D printing and freeze-drying, and successfully applied them to the integrated repair of osteochondral defects. In this study, 3D conductive scaffolds with a multilevel structure were successfully fabricated by combining low-temperature deposition 3D printing technology with nanotechnology. The pressure value of ink with high viscosity should be set higher, or the moving speed of nozzle should be set lower, appropriately. When using ink with low viscosity, the pressure value should be set lower, appropriately or the moving speed of nozzle should be accelerated. For the ink containing 15 wt% or 20 wt% T-PPy, the extrusion speed of shear thickening should be avoided as far as possible when printing, and the parameters of high pressure and high speed should be used for printing. 

SEM images show scaffolds containing S-PPy have larger surface area, more agglomeration, and more uneven dispersion in the matrix at the same concentration compared with T-PPy, which will result in more microfilament porosity. In order to characterize the electrical properties of conductive scaffolds, the same process was used to prepare conductive scaffolds filament and characterize the conductivity of conductive scaffold filament. The results showed that the conductivity of the conductive scaffold containing 30 wt% S-PPy can be close to 10^−4^ S/cm, which has similar conductivity to the physiological tissue. Comparing with S-PPy, T-PPy with the same concentration have higher conductivity, which may be attributed to the easier intertwining and contact between adjacent T-PPy, resulting in seepage at lower concentration. Previous studies have shown that the introduction of a certain amount of tubular nanoparticles will enhance the mechanical strength of 3D printing scaffolds [32]. Herein, the concentration of PLLA decreased with the increase of the concentration of PPy nanoparticles, so when the concentration of PPy is excessive, the mechanical strength of 3D scaffolds decreases, which may be used to reasonably explain the phenomenon that the mechanical properties of scaffold containing T-PPy vary with its concentration. Furthermore, 3D scaffolds are not suitable for periosteum, perineurium, and skin repair, in which 2D scaffolds should be prepared. However, the traditional preparation technology such as solvent casting, produces 2D thin film with bubbles and unequal thickness defects frequently. In this study, we creatively integrated the advantages of 3D printing and solvent casting and successfully prepared 2D conductive films scaffold with no bubbles, uniform thickness, and good structural stability. Similar to solvent casting, the surface roughness of the film containing PPy nanoparticles was higher than that of the pure PLLA film. The conductivity of the films reached 10^−4^ S/cm, which has similar conductivity to that of the physiological tissues. Furthermore, the tensile strength of the films increased with the addition of T-PPy, which may be attributed to the entanglement between the T-PPy and PLLA molecular chains.

The cytotoxicity of materials used in tissue engineering scaffolds should be particularly low. The composite materials with spherical and tubular PPy nanoparticles were demonstrated to be cytocompatible, which indicated their applicability as tissue engineering scaffolds.

## 5. Conclusions

Conductive scaffolds with conductivity similar to natural tissues were successfully fabricated by combining 3D printing with nanotechnology in this study. Specifically, for the construction of 3D tissue engineering scaffolds, 3D conductive scaffold with macrocontrollable pore (~100 μm) and microfilament pore (~10 μm) was successfully fabricated by combining extrusion-based low-temperature deposition 3D printing with freeze-drying. For the construction of 2D tissue engineering scaffolds, 2D nanocomposite conductive film scaffold with uniform thickness, no bubbles, and good structural stability were successfully fabricated by creatively combining extrusion-based 3D printing with solvent casting. In conclusion, the 3D and 2D tissue engineering scaffolds prepared in this study have been successfully constructed to optimize the physiological microenvironment, which can be adjusted in a wide range to adapt to the repair and regeneration of different physiological tissues. Next, we will further study the biological properties of the tissue engineering scaffolds in vivo and in vitro. In particular, we will study the effect of conductivity on the differentiation of stem cells.

## Figures and Tables

**Table 1:** Composition and proportion of three-dimensional (3D) printing ink.

Sample (C_PPy_–C_ink_-S/T)	m_PPy(T)_ (g)	m_PPy(S)_ (g)	m_PLLA_ (g)	V_1,4-dioxane_ (mL)	C_PPy_	C_ink_
0%–10%	0	0	1	10	0	10%
5%–10%-S	0	0.05	0.95	10	5%	10%
10%–10%-S	0	0.1	0.9	10	10%	10%
15%–10%-S	0	0.15	0.85	10	15%	10%
50%–10%-S	0	0.5	0.5	10	50%	10%
5%–10%-T	0.05	0	0.95	10	5%	10%
10%–10%-T	0.1	0	0.9	10	10%	10%
15%–10%-T	0.15	0	0.85	10	15%	10%
20%–10%-T	0.2	0	0.8	10	20%	10%
0%–15%	0	0	1.5	10	0	15%
5%–15%-S	0	0.075	1.425	10	5%	15%
10%–15%-S	0	0.15	1.35	10	10%	15%
15%–15%-S	0	0.225	1.275	10	15%	15%
30%–15%-S	0	0.45	1.05	10	30%	15%
5%–15%-T	0.075	0	1.425	10	5%	15%
10%–15%-T	0.15	0	1.35	10	10%	15%
15%–15%-T	0.225	0	1.275	10	15%	15%
20%–15%-T	0.3	0	1.2	10	20%	15%
30%–15%-T	0.45	0	1.05	10	30%	15%

## References

[1] Guo B., Ma P.X. (2018). Conducting Polymers for Tissue Engineering. Biomacromolecules.

[2] Thrivikraman G., Mallik P.K., Basu B. (2013). Substrate conductivity dependent modulation of cell proliferation and differentiation in vitro. Biomaterials.

[3] Qian Y., Song J., Zhao X., Chen W., Ouyang Y., Yuan W., Fan C. (2018). 3D Fabrication with Integration Molding of a Graphene Oxide/Polycaprolactone Nanoscaffold for Neurite Regeneration and Angiogenesis. Adv. Sci..

[4] Liang S., Zhang Y., Wang H., Xu Z., Chen J., Bao R., Tan B., Cui Y., Fan G., Wang W. (2018). Paintable and Rapidly Bondable Conductive Hydrogels as Therapeutic Cardiac Patches. Adv. Mater..

[5] Gajendiran M., Choi J., Kim S.J., Kim K., Shin H., Koo H.J., Kim K. (2017). Conductive biomaterials for tissue engineering applications. J. Ind. Eng. Chem..

[6] Jiang L., Wang Y., Liu Z., Ma C., Yan H., Xu N., Gang F., Wang X.M., Zhao L., Sun X. (2019). 3D Printing and Injectable Conductive Hydrogels for Tissue Engineering Application. Tissue Eng. Part B Rev..

[7] Bini T.B., Gao S., Tan T.C. (2004). Electrospun poly(L-lactide-co-glycolide) biodegradable polymer nanofibre tubes for peripheral nerve regeneration. Nanotechnology.

[8] Zhou J., Wang Y., Cheng L., Wu Z., Sun X.D., Peng J. (2016). Preparation of polypyrrole-embedded electrospun poly(lactic acid) nanofbrous scaffolds for nerve tissue engineering. Neural Regen. Res..

[9] Zhou J., Cheng L., Sun X., Wang X., Jin S., Li J., Wu Q. (2016). Neurogenic differentiation of human umbilical cord mesenchymal stem cells on aligned electrospun polypyrrole/polylactide composite nanofibers with electrical stimulation. Front. Mater. Sci..

[10] Shu B., Sun X., Liu R., Jiang F., Yu H., Xu N., An Y. (2019). Restoring electrical connection using a conductive biomaterial provides a new therapeutic strategy for rats with spinal cord injury. Neurosci. Lett..

[11] Shu B., Liu X.B., Zhou J.F., Huang H., Wang J.Y., Sun X.D., Qin C., An Y.H. (2019). Polypyrrole/polylactic acid nanofibrous scaffold cotransplanted with bone marrow stromal cells promotes the functional recovery of spinal cord injury in rats. CNS Neurosci. Ther..

[12] Menčík P., Přikryl R., Stehnová I., Melčová V., Kontárová S., Figalla S., Alexy P., Bočkaj J. (2018). Effect of Selected Commercial Plasticizers on Mechanical, Thermal, and Morphological Properties of Poly (3-hydroxybutyrate)/Poly (lactic acid)/Plasticizer Biodegradable Blends for Three-Dimensional (3D) Print. Materials.

[13] Pekkanen A.M., Mondschein R.J., Williams C.B., Long T.E. (2017). 3D Printing Polymers with Supramolecular Functionality for Biological Applications. Biomacromolecules.

[14] Farahani R.D., Dubé M., Therriault D. (2016). Three-dimensional printing of multifunctional nanocomposites: Manufacturing techniques and applications. Adv. Mater..

[15] Sensharma P., Madhumathi G., Jayant R.D., Jaiswal A.K. (2017). Biomaterials and cells for neural tissue engineering: Current choices. Mater. Sci. Eng. C Biol. Appl..

[16] Qian Y., Zhao X., Han Q., Chen W., Li H., Yuan W. (2018). An integrated multi-layer 3D-fabrication of PDA/RGD coated graphene loaded PCL nanoscaffold for peripheral nerve restoration. Nat. Commun..

[17] Qian Y., Song J., Zheng W., Zhao X., Ouyang Y., Yuan W., Fan C. (2018). 3D Manufacture of Gold Nanocomposite Channels Facilitates Neural Differentiation and Regeneration. Adv. Funct. Mater..

[18] Lee S.J., Zhu W., Nowicki M., Lee G., Heo D.N., Kim J., Zuo Y.Y., Zhang L.G. (2018). 3D printing nano conductive multi-walled carbon nanotube scaffolds for nerve regeneration. J. Neural Eng..

[19] Dai T., Yang X., Lu Y. (2006). Controlled growth of polypyrrole nanotubule/wire in the presence of a cationic surfactant. Nanotechnology.

[20] De Santis R., D’Amora U., Russo T., Ronca A., Gloria A., Ambrosio L. (2015). 3D fibre deposition and stereolithography techniques for the design of multifunctional nanocomposite magnetic scaffolds. J. Mater. Sci. Mater. Med..

[21] Domingos M., Gloria A., Coelho J., Bartolo P., Ciurana J. (2017). Three-dimensional printed bone scaffolds: The role of nano/micro-hydroxyapatite particles on the adhesion and differentiation of human mesenchymal stem cells. Proc. Inst. Mech. Eng. H.

[22] Ho C.M.B., Mishra A., Lin P.T.P., Ng S.H., Yoeng W.Y., Kim Y.J., Yoon Y.J. (2017). 3D printed polycaprolactone carbon nanotube composite scaffolds for cardiac tissue engineering. Macromol. Biosci..

[23] Spath S., Drescher P., Seitz H. (2015). Impact of particle size of ceramic granule blends on mechanical strength and porosity of 3D printed scaffolds. Materials.

[24] Huang B., Vyas C., Roberts I., Poutrel Q.A., Chiang W.H., Blaker J.J., Huang Z., Bártolo P. (2019). Fabrication and characterisation of 3D printed MWCNT composite porous scaffolds for bone regeneration. Mater. Sci. Eng. C Mater. Biol. Appl..

[25] Therriault D., White S.R., Lewis J.A. (2003). Chaotic mixing in three-dimensional microvascular networks fabricated by direct-write assembly. Nat. Mater..

[26] Gang F.L., Yan H., Ma C.Y., Jiang L., Gu Y., Liu Z., Zhao L., Wang X., Zhang J., Sun X. (2019). Robust magnetic double-network hydrogels with self-healing, MR imaging, cytocompatibility and 3D printability. Chem. Comm..

[27] Therriault D., Shepherd R.F., White S.R., Lewis J.A. (2005). Fugitive inks for Direct-Write assembly of Three-Dimensional microvascular networks. Adv. Mater..

[28] Yu K., Cao H., Qian K., Sha X., Chen Y. (2012). Shear-thickening behavior of modified silica nanoparticles in polyethylene glycol. J. Nanopart. Res..

[29] Zhang T., Zhang H., Zhang L., Jia S., Liu J., Xiong Z., Sun W. (2017). Biomimetic design and fabrication of multilayered osteochondral scaffolds by low-temperature deposition manufacturing and thermal-induced phase-separation techniques. Biofabrication.

[30] Lai Y., Li Y., Cao H., Long J., Wang X., Li L., Li C., Jia Q., Teng B., Tang T. (2019). Osteogenic magnesium incorporated into PLGA/TCP porous scaffold by 3D printing for repairing challenging bone defect. Biomaterials.

[31] Lin S., Cui L., Chen G., Huang J., Yang Y., Zou K., Lai Y., Wang X., Zou L., Wu T. (2019). PLGA/β-TCP composite scaffold incorporating salvianolic acid B promotes bone fusion by angiogenesis and osteogenesis in a rat spinal fusion model. Biomaterials.

[32] Woodard J.R., Hilldore A.J., Lan S.K., Park C.J., Morgan A.W., Eurell J.A., Clark S.G., Wheeler M.B., Jamison R.D., Wagoner Johnson A.J. (2007). The mechanical properties and osteoconductivity of hydroxyapatite bone scaffolds with multi-scale porosity. Biomaterials.

