# Correction: Ma et al. 3D Printing of Conductive Tissue Engineering Scaffolds Containing Polypyrrole Nanoparticles with Different Morphologies and Concentrations. *Materials* 2019, *12*, 2491

**DOI:** 10.3390/ma19143062

**Published:** 2026-07-16

**Authors:** Chunyang Ma, Le Jiang, Yingjin Wang, Fangli Gang, Nan Xu, Ting Li, Zhongqun Liu, Yongjie Chi, Xiumei Wang, Lingyun Zhao, Qingling Feng, Xiaodan Sun

**Affiliations:** 1 School of Earth Sciences and Resources, China University of Geosciences (Beijing), Beijing 100083, China; 2 State Key Laboratory of New Ceramics and Fine Processing, School of Materials Science and Engineering, Tsinghua University, Beijing 100084, China; 3 Key Laboratory of Advanced Materials of Ministry of Education of China, School of Materials Science and Engineering, Tsinghua University, Beijing 100084, China

## Figure Legend

In the original publication [1], there was a mistake in the legends for Figures 1 and 6. The correct legends appear below:

**Figure 1.** SEM images (**a**) and TEM images (**b**) of tubular PPy nanoparticles (T-PPy), SEM images (**c**) and TEM images (**d**) of spherical PPy nanoparticles (S-PPy). Scale bar is 200 nm.
**Figure 6.** SEM images of pure poly-l-lactide (PLLA) film and films containing 10 wt% T-PPy and S-PPy, (**a**–**c**) physical images of three films, scale bar is 1 cm (pure, T-PPy, and S-PPy, in turn)), (**d**–**f**) SEM images of three films at 104 magnification, scale bar is 200 μm (10 wt% PPy, T-PPy, and S-PPy, in turn), (**g**–**i**) SEM images of three films at 10,000 magnification, and scale bar is 1 μm (10 wt% PPy, T-PPy, and S-PPy, in turn).

## Error in Figure

In the original publication [1], there was a mistake in Figure 3. The corrected Figure 3 is shown below:

Correspondingly, the citation of Figure 3 in Section 3.3 paragraph 1 has been corrected. The sentence “and scaffold containg S-PPy appeared to be more porous” should be deleted.

The authors state that the scientific conclusions are unaffected. This correction was approved by the Academic Editor. The original publication has also been updated.

## Figures and Tables

**Figure 3:**
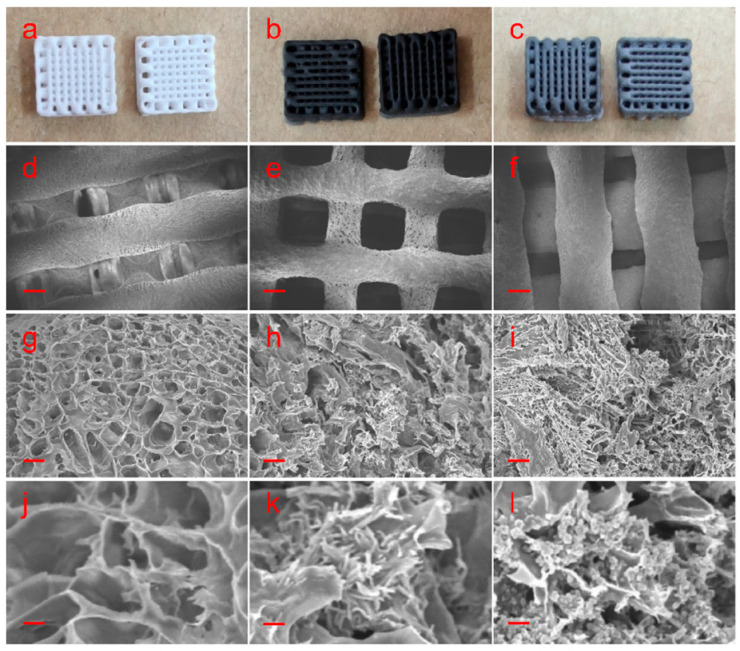
Optical and SEM images of 3D conductive scaffolds containing 10 wt% T-PPy or S-PPy prepared by combining 3D printing and freeze-drying. (**a**–**c**) Optical images of 3D conductive scaffolds (pure, tubular, and spherical in turn), (**d**–**f**) SEM images of the scaffolds at the magnification of 26, scale bar is 200 μm, (pure, tubular, and spherical in turn), (**g**–**i**) SEM images of the scaffolds at the magnification of 1000, scale bar is 10 μm, (**j**–**l**) SEM images of the scaffolds at the magnification of 10,000, scale bar is 1 μm.
